# Analysis of Xylose Operon from *Paenibacillus polymyxa* ATCC842 and Development of Tools for Gene Expression

**DOI:** 10.3390/ijms23095024

**Published:** 2022-04-30

**Authors:** Zilong Wang, Yakun Fang, Yi Shi, Yu Xin, ZhengHua Gu, Ting Yang, Youran Li, Zhongyang Ding, Guiyang Shi, Liang Zhang

**Affiliations:** 1 National Engineering Research Center for Cereal Fermentation and Food Biomanufacturing, Jiangnan University, Wuxi 214122, China; 7180201060@stu.jiangnan.edu.cn (Z.W.); 7170201031@stu.jiangnan.edu.cn (Y.F.); shiyi0621@jiangnan.edu.cn (Y.S.); yuxin@jiangnan.edu.cn (Y.X.); guzhenghua1975@163.com (Z.G.); liyouran@jiangnan.edu.cn (Y.L.); zyding@jiangnan.edu.cn (Z.D.); gyshi@jiangnan.edu.cn (G.S.); 2 Jiangsu Provincial Engineering Research Center for Bioactive Product Processing, Jiangnan University, 1800 Lihu Avenue, Wuxi 214122, China; 3 Wuxi Food Safety Inspection and Test Center, Wuxi 214142, China; 7160201018@vip.jiangnan.edu.cn; 4 Technology Innovation Center of Special Food for State Market Regulation, Wuxi 214142, China

**Keywords:** *Paenibacillus polymyxa*, xylose operon, inducible expression system

## Abstract

With numerous industrial applications, *Paenibacillus polymyxa* has been accepted as the candidate of the cell factory for many secondary metabolites. However, as the regulatory expression elements in *P. polymyxa* have not been systematically investigated, genetic modification on account of a specific metabolism pathway for the strain is limited. In this study, a xylose-inducible operon in the xylan-utilizing bacterium ATCC842 was identified, and the relative operon transcription was increased to 186-fold in the presence of xylose, while the relative enhanced green fluorescent protein (eGFP) fluorescence intensity was promoted by over four-fold. By contrast, glucose downregulated the operon to 0.5-fold that of the control. The binding site of the operon was “ACTTAGTTTAAGCAATAGACAAAGT”, and this can be degenerated to “ACTTWGTTTAWSSNATAVACAAAGT” in *Paenibacillus* spp., which differs from that in the *Bacillus* spp. xylose operon. The xylose operon binding site was transplanted to the constitutive promoter P*shuttle-09*. The eGFP fluorescence intensity assay indicated that both the modified and original P*shuttle-09* had similar expression levels after induction, and the expression level of the modified promoter was decreased to 19.8% without induction. This research indicates that the operon has great potential as an ideal synthetic biology tool in *Paenibacillus* spp. that can dynamically regulate its gene circuit strength through xylose.

## 1. Introduction

*Paenibacillus polymyxa* is a biological control and plant-growth-promoting bacterium that produces numerous primary and secondary metabolites [1,2,3,4]. This bacterium’s nitrogen fixation ability and production of flavonoids and terpenoids [5] could help to promote the growth of plants. Numerous polyketides, lipopeptides [6], and alkaloids [7] produced by *P. polymyxa* have been characterized and broadly applied in the agriculture, food, and pharmaceutical industries. Various types of exopolysaccharides produced by *P. polymyxa* using different carbons [8] could be widely used in coatings and cosmetics.

To obtain these valuable products more efficiently, technologies involving fermentation engineering and genetic and metabolic engineering have been introduced in recent decades. For instance, Polymyxin E is an antibiotic with therapeutic efficacy against Gram-negative bacterial pathogens, and Yu et al. [9,10] improved the yield of it from *P. polymyxa* by optimizing the culture medium. Zhang et al. [11] improved R,R-2,3-butanediol biosynthesis and obtained a product of ultrahigh optical purity via metabolic engineering. The CRISPR-Cas9 knockout system has also been introduced for research on exopolysaccharides in *P. polymyxa* [12]. However, due to the lack of valid expression elements, the foregoing studies mainly applied integration expression techniques in the research on *P. polymyxa*. To improve the expression efficiency, using a free-expression system with relatively higher copy numbers might be a promising strategy.

Certain promoters derived from other bacterial species have been integrated into *P. polymyxa* successfully [12,13]. Nevertheless, exogenous inducible promoters may be incompatible and are at risk of leakage [14], while no endogenous promoters have currently been mined or applied. Among endogenous promoters, constitutive promoters are suggested to have higher expression levels in the absence of any inducer than any other inducible promoters. On the other hand, without generating products that impede bacterial growth in the early stages, inducible promoters could be applied for the expression of certain toxic proteins and thus improve the application potential of *P. polymyxa* if an appliable inducible expression system were constructed for this strain.

Via the unique regulatory mode, inducible expression systems can be activated with high expression levels by chemical or environmental induction or by autoinduction [15,16,17]. Chemically induced expression systems are preferred in industrial production due to their cost efficiency. For practical applications, common inducers include arabinose [18], mannose [19], fructose [20], galactose [21], xylose [22], lactose [23], maltose [24], sucrose [25], or other sugars in sugar-inducible systems as well as sorbitol [26], mannitol [27], methanol [28], and ethanol [29] in alcohol-inducible systems. Characterized by the advantages of high expression levels and strong inducer specificity, the xylose-inducible expression system has been constructed and verified in *Bacillus subtilis*, *Bacillus licheniformis*, *Bacillus megaterium*, and *Bacillus coagulans* [22,30,31,32]. The utilization of xylose in *Bacillus* spp. requires xylose isomerase and xylulose kinase, which are encoded by *xylA* and *xylB*, respectively. Both genes are regulated at the transcription level by a xylose-responsive repressor ROK family protein encoded by *xylR*. When xylose is deficient, XylR binds the operator, prevents RNA polymerase transcription, and regulates P*xylR* and P*xylAB*. The XylR binding site contains GTTT N7 AAAC [33,34], which is identified by the helix–turn–helix (HTH) domain in ROK family members. In the latter, the HTH domain corresponds to the N-terminal DNA-binding domain. The Arg side chain interacts with the GC while the Lys side chain interacts with the TA beside it [31]. Arg- and Gly-rich linkers help ROK family proteins to read the electrostatic potential of the minor grooves [35,36]. The xylose operator is controlled by carbon catabolite repression (CCR) using a catabolite-responsive element (cre). The latter is a characteristic cis-active CCR sequence with which catabolite control protein A (CcpA) interacts. This assemblage negatively regulates the transcription of genes controlled by sugar-inducible operons in the presence of carbon sources, such as glucose [37].

For *P. polymyxa*, although this bacterium can grow with xylan and produces 2,3-butanediol acid using xylose as the carbon source, the pathway and operator associated with xylose metabolism in this strain have not been identified. To construct valid inducible expression systems in *P. polymyxa*, in the present study, we elucidated the sequences of *xylA*, *xylB*, and *xylR*, and verified the relationships between their transcriptional intensity and the presence of xylose or glucose as a carbon source. A novel inducible promoter was generated and verified herein through the verification of promoter expression intensity by green fluorescent protein (eGFP), analyzation of the xylose operon structure for the confirmation of the XylR binding site, and transplantation of the bases in the repressor protein binding site to the constitutive promoter after identification and localization. With the introduction of the xylose-inducible promoter in *P. polymyxa*, the current study provides a promising expression element with the intention to extend the application potential of this certified biosafety microorganism.

## 2. Results

### 2.1. Analysis of Xylose Operon Elements and Function in P. polymyxa

**Figure 1:**
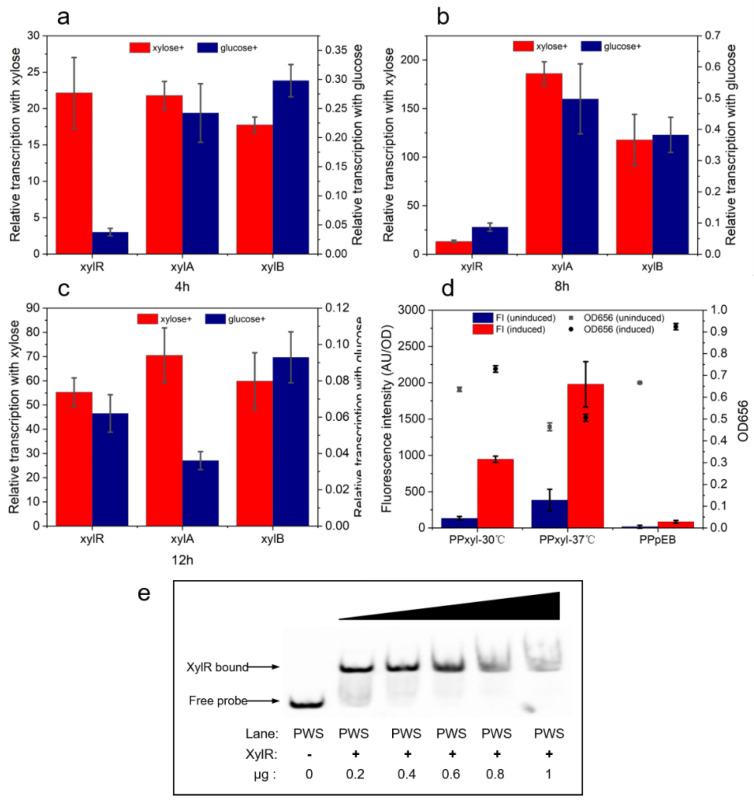
Experimental evidence for regulation of the predicted operon by xylose. Transcriptional strengths of *xylR*, *xylA*, and *xylB* after 4 h (**a**), 8 h (**b**), and 12 h (**c**) of culture with xylose or glucose. The fluorescence intensity level of eGFP (**d**) expressed by the predicted xylose-inducible promoter in the presence of 2% xylose. ATCC842 containing pHY300PLK-eGFP was the control and is labeled as PPpEB. The EMSA assay result (**e**) shows the binding capacity of XylR to the predicted promoter.

The whole-genome sequence of *P. polymyxa* ATCC842 has been published in the National Center for Biotechnology Information (NCBI) database (GenBank: CP049783.1). The analysis revealed that the presence of the predicted xylose operon encoded three putative open reading frames. The encoded proteins were annotated as ROK family transcriptional regulator (CP049783.1, 2816955-2818124), xylose isomerase (CP049783.1, 2818260-2819576), and xylulokinase (CP049783.1, 2819633-2821156). The gene direction of the hypothetical transcriptional regulator differed from those of the other two genes, and the latter were xylose metabolism proteins. Hence, there was a bidirectional promoter between these two reverse genes. This bidirectional starting structure resembled those of the xylose operons from *B. subtilis, B. licheniformis,* and *B. coagulans*. ROK family proteins are associated with sugar metabolism and include a sugar kinase and a sugar-responsive repressor. To identify the function of this ROK family protein, we aligned its protein sequence with that of XylR from *B. subtilis* (AL009126.3, 1890512-1891666) and *B. licheniformis* (CP000002.3, 3876248-3877411), which regulate xylose utilization, and with that of Lmo0178 (NC_003210.1, 177924-179138) from *Listeria monocytogenes*, which regulates α-glucan decomposition (Figure S1). The alignment results reveal a 38.62% similarity. Two structures (HTH domain and linker) of ROK family proteins, which play important roles in DNA binding [36], have been identified in the predicted regulator with similarities of 66.67% and 65%, respectively. As such, these DNA-binding structures were suggested to be within the ROK family protein, whose binding site may be located before *xylA* to control XylA and XylB expression. To verify this hypothesis, qPCR was applied to measure the transcription intensity and verify the relationship between xylose and the transcription of the genes controlled by the predicted xylose operon. The results (Figure 1a) show that *xylR*, *xylA*, and *xylB* were significantly upregulated (22-fold, 21-fold, and 17-fold, respectively) after 4 h of culture with xylose. The transcription of all three genes continued to be upregulated (13-fold, 186-fold, and 117-fold, respectively) after 8 h of culture with xylose (Figure 1b). After 12 h of culture with xylose (Figure 1c), the transcription rates of the genes declined whilst still being identified as upregulated (55-fold, 71-fold, and 60-fold, respectively). The *xylA* and *xylB* transcription intensity initially increased and then decreased with xylose consumption, while *xylR* transcription intensity decreased at 8 h whereas it was at its maximum for *xylA* and *xylB* at that time. When culturing with glucose, *xylR*, *xylA*, and *xylB* were significantly downregulated (0.04-fold, 0.24-fold, and 0.29-fold, respectively) after 4 h of culture, and more so after 8 h of culture (0.09-fold, 0.5-fold, and 0.38-fold, respectively). After 12 h, the transcription intensity levels of these genes were only 0.06-fold, 0.04-fold, and 0.09-fold, respectively.

The xylose operon expression efficiency was then evaluated by using the promoter to control eGFP expression. After an induction of 8 h, the eGFP fluorescence intensity levels were 947 AU/OD and 1980 AU/OD for *P. polymyxa* cultured at 30 °C and 37 °C, respectively (Figure 1d). The fluorescence intensity of the cells cultured at 37 °C was significantly higher than that for cells cultured at 30 °C. However, when comparing these two culture temperatures, the OD_656_ was lower for *P. polymyxa* cultured at 37 °C.

In an electrophoretic mobility shift assay (EMSA) experiment, the ROK family protein was identified as a candidate for regulating the bidirectional promoter, which was confirmed by a shifted band in the lane with the biotinylated promoter as a probe. As shown in Figure 1e, the ratio of the shifted band was increased when the concentration of XylR improved in the lane, suggesting that XylR can bind to the promoter.

As shown in this section, the ROK family transcriptional regulator may in fact bind the xylose operon. The predicted ROK family protein and the bidirectional promoter comprised the xylose operon in *P. polymyxa.* Moreover, xylose was found to promote the operon whereas glucose inhibited it.

### 2.2. Efficient Expression of Protein by PPxyl in P. polymyxa

**Figure 2:**
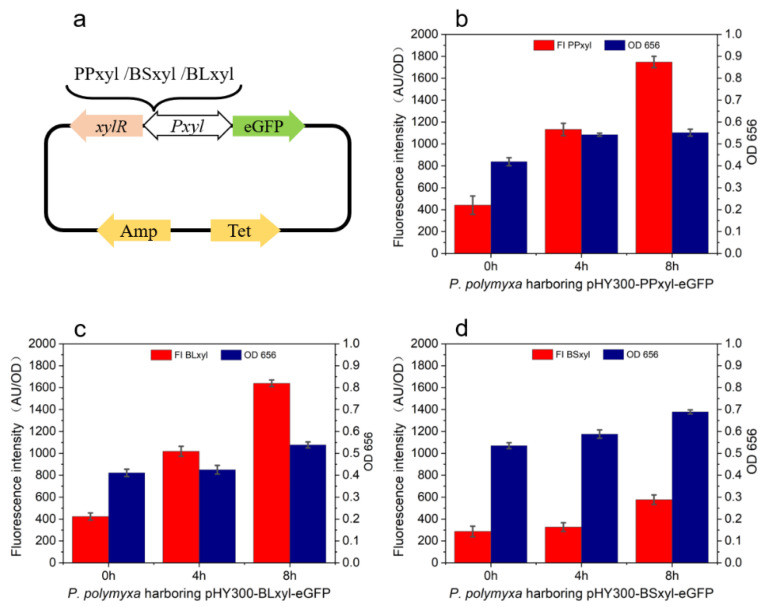
Fluorescence intensity levels of *P. polymyxa* harboring plasmids bearing xylose operons from different species. Construction of plasmids harboring *eGFP* and xylose operons from different species (**a**)**.** The OD_656_ and fluorescence intensity levels of eGFP expressed by *P. polymyxa* (**b**)*, B. licheniformis* (**c**), *and* *B. subtilis* (**d**) xylose-induced operon.

To compare the expression efficiency levels of the xylose operons in different species, *P. polymyxa* with the plasmid pHY300PLK harboring the xylose operon from *B. subtilis*, *B. licheniformis*, and *P. polymyxa* was induced with xylose, and its fluorescence intensity was measured. The corresponding plasmid construction scheme is shown in Figure 2a. With xylose as the inducer, the fluorescence intensity levels of PP*xyl*, Bl*xyl*, and BS*xyl* after an induction of 4 h were 1132, 1019, and 128, respectively, and these levels were 1748, 1639, and 577, respectively, after 8 h. For the biomass, the OD_656_ was measured as 0.54, 0.42, and 0.58, respectively, after 4 h of induction, while it was 0.55, 0.53, and 0.69, respectively, after 8 h of induction. The growth rates of *P. polymyxa* containing plasmids harboring PP*xyl* and BL*xyl* were lower than those of the cells carrying plasmids harboring BS*xyl*. The fluorescence intensity levels of the cells harboring PP*xyl* or BL*xyl* were significantly higher than those of the cells harboring BS*xyl* (Figure 2b–d). With reflecting the promoter expression efficiency by measuring the fluorescence intensity, the biomass growth rate was observed to decrease with the increasing expression efficiency due to the impacts brought about by eGFP on cell growth.

### 2.3. Similar Xylose Operons from P. polymyxa with Different Binding Sites

**Figure 3:**
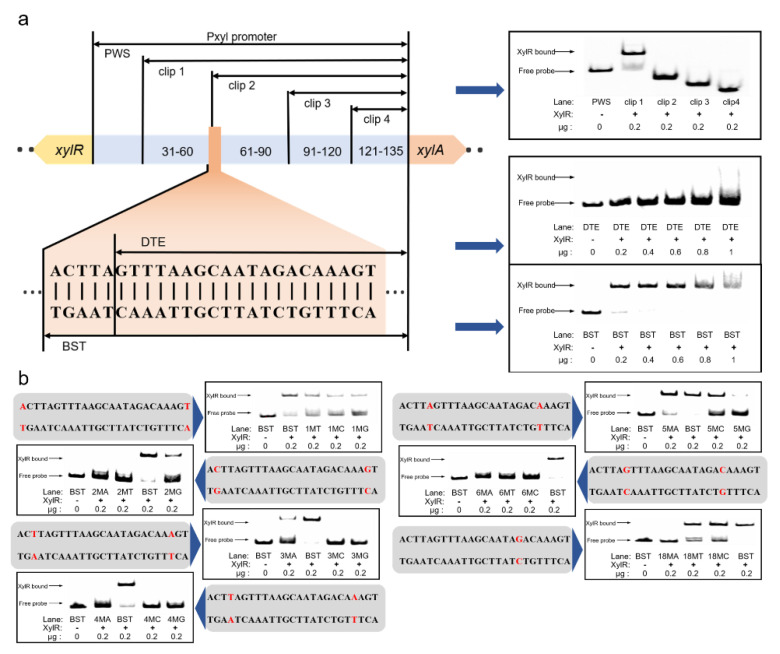
EMSA experiment localizing the XylR binding site in the xylose operon. (**a**) EMSA results of sequence with full and damaged binding sites. Top half shows the XylR protein binding to the truncated promoter confirmed by EMSA; bottom half shows the specific presumed binding site sequence. (**b**) The mutation of seven bases in the binding site affected the XylR protein binding ratio.

**Figure 4:**
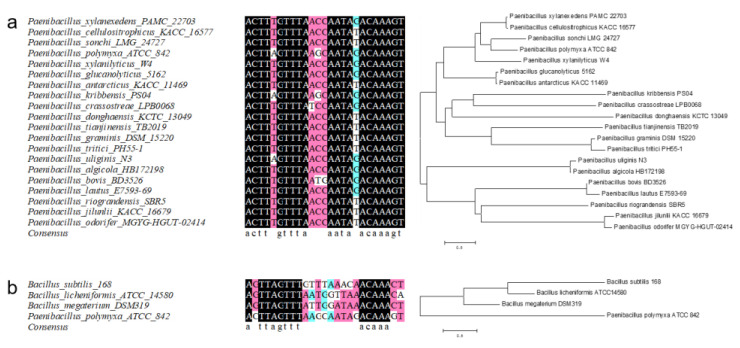
Homology analysis and phylogenetic tree construction of XylR binding sites in *P. polymyxa* and other bacteria. A comparison of XylR binding site from *P. polymyxa* and other *Paenibacillus* spp. is shown in (**a**), and a comparison of XylR binding site from *P. polymyxa* and other *Bacillus* spp. is shown in (**b**).

To elucidate the structure of the bidirectional promoter, the corresponding sequence was aligned with those of other *P. polymyxa* sequences in the NCBI database (Figure S2). The results indicate that the −10 and −35 of P*xylR* and P*xylAB* are located at 36–41 bp and 59–64 bp and at 92–97 bp and 69–74 bp, respectively. EMSA was then applied to investigate the binding relationship between the xylose promoter and XylR in vitro. The promoter was gradually truncated into the fragments PP*xyl*−30 bp, −60 bp, −90 bp, and −120 bp. The XylR bound the fragment Clip 1 but could not bind fragments Clip 2–Clip 4, which truncated over 60 bp (Figure 3a). Compared with the binding sites in *B. subtilis* and *B. licheniformis*, the *P. polymyxa* binding site may contain a GTTT-to-AAAC structure. A similar sequence, namely GTTTAAGCAATAGAC, was found in the conserved area at 59–73 bp. The similar sequences GAATCGTTTTTGTAAAC, GTTTTTGTAAAC, and GTTTTTGTAAACGTTTAC were detected in the conserved area at 92–115 bp. However, the sequence at 57–70 bp occurred only in Clip 1 and was assumed to be the XylR binding site. To confirm the complete binding site sequence, the palindrome sequence AAACTTAGTTTAAGCAATAGACAAAGTTT was detected in the ATCC842 P*xyl* promoter harboring the aforementioned 17 bp structure. Nevertheless, base T at 79 bp is not conserved in all *P. polymyxa* strains. Hence, the binding site sequence is predicted to be ACTTAGTTTAAGCAATAGACAAAGT. To verify this conjecture, another two fragments containing the full predicted site (BST) and damaged site (DTE) were introduced in an EMSA experiment (Figure 3a). The XylR protein in the molar ratio of 20.9–104.4 between the protein and the probe (XylR concentration range 0.02–0.1 g/L) bound the fragment with the full site but not the fragment without ACTTA. Hence, ACTTA seems to play a vital role in protein binding. As shown in Figure 3b, the significance of the six peripheral bases at the site was further confirmed. In the BST fragment, which bound the XylR protein at an original ratio of 70%, each base pair was mutated to another three types. After modifying the edge bases A/T at 54 bp and 78 bp to T/A, it was found that the binding ratio was reduced to 30%. When the bases were changed to C/G and G/C, the binding ratios were lowered to 19.8% and 18.8%, respectively. The binding ratio was changed to 21.3% if the second bases (C/G) at 55 bp and 77 bp were changed to G/C, whilst the ratio became 0% after changing the fragments to A/T or T/A. Regarding the T/A third bases at 56 bp and 76 bp, the binding ratio was 16.9% when mutating to A/T. When they were mutated from C/G to G/C, no binding occurred. This phenomenon was also found for the fourth T/A bases at 57 bp and 75 bp, in which we only observed a binding ratio of 3.5% when these bases were mutated to A/T. When the fifth A/A bases at 58 bp and 74 bp were mutated to G/C, C/G, and T/T, the binding ratios were 10.5%, 32.8%, and 55%, respectively. When the sixth G/C bases were mutated, the binding ratios for the fragments with A/T, C/G, and T/A mutations were 6.4%, 0%, and 0%, respectively. Therefore, the four edge bases from GTTT to AAAC and the first bases (G/C) seem to play important roles in XylR protein binding, whereas the fourth bases were only loosely compatible. In addition, the base G at 71 bp, which broke the palindrome, was tested as well. It was found that the binding ratios were 52.1% and 63.5% after mutating the base to T and C, respectively. The binding ratio was then observed to be only 1.9% when the palindrome was repaired by a point mutation to A. To evaluate the effect of the bases out of the predicted site, the first (53 bp and 79 bp) and second (54 bp and 80 bp) A/T bases in the periphery were mutated to T/A, G/C, and C/G. The corresponding binding ratios of each mutation for the first base were 61.3%, 64.5%, and 50.8%, while they were 65.4%, 60.8%, and 57.6% for the second base (Figure S3). To further verify the universality of the binding site, we compared the binding site with another 19 kinds of xylose operons from *Paenibacillus* spp. The *Paenibacillus* spp. xylose operons were predicted using the XylA protein sequence from *P. polymyxa*. The alignment results are shown in Figure 4a. The binding site of XylR is highly conservative in *Paenibacillus* spp.; the sequence had 20 bp identical to other *Paenibacillus* spp. The sequence could degenerate as ACTTWGTTTAWSSNATAVACAAAGT, and the fifth base and eighteenth base were also T/A and G/T in *Paenibacillus* spp., as previously mentioned. In order to determine the scope of application of the binding site, we also compared the binding site with typical binding sites in *Bacillus* spp. (Figure 4b). The results show that only 13 bp were identified in *Bacillus* spp.

### 2.4. Promoter Transformation Based on Pxyl Binding Site

**Figure 5:**
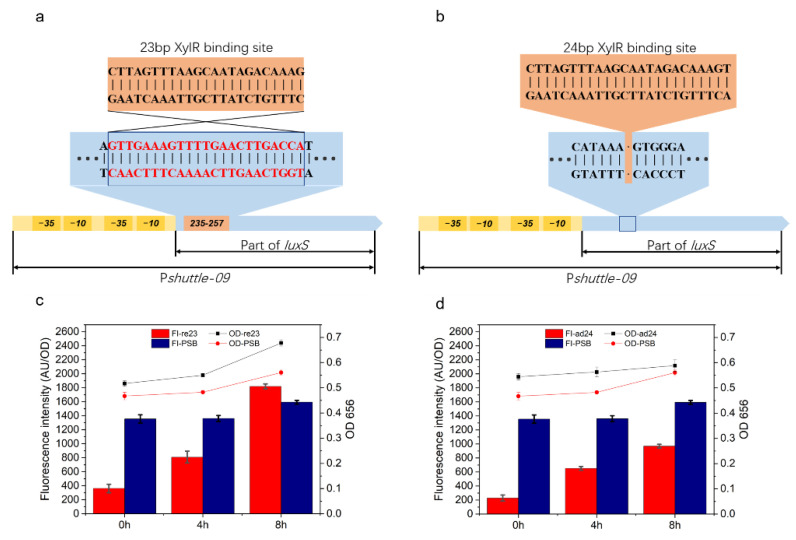
Constructions and fluorescence intensities of modified P*shuttle-09* promoters. (**a**) Construction of Psr23; the 23 bp XylR binding site was introduced into P*shuttle-09* by replacing 23 bp sequence. (**b**) Construction of Psa24; the 24 bp XylR binding site was introduced into P*shuttle-09* by inserting it between 303 bp and 304 bp bases. The fluorescence intensities of *P. polymyxa* carrying plasmid with Psr23 and Psa24 are shown in (**c**) and (**d**).

In this study, the possible available types of inducible promoters in *P. polymyxa* were investigated, and the effect of the XylR binding site on the corresponding promoter was also determined. In order to expand the application scope of this regulation, we transformed the binding site into a constitutive promoter, and the efficiencies of the modified promoters were evaluated by measuring the fluorescence intensity of eGFP. The P*shuttle-09* promoter is a hybrid constitutive promoter constructed with two core regions and an 87 bp-truncated *LuxS* sequence. In order to avoid damage to the core region of this promoter and frameshift mutation, the modification was performed on the truncated *LuxS* gene sequence by replacing GTTGAAAGTTTTGAACTTGACCA with the 23 bp sequence CTTAGTTTAAGCAATAGACAAAG at 235–257 bp located between bases A and T. The region was then designated as Psr23 (Figure 5a). In another experiment, a 24 bp sequence (CTTAGTTTAAGCAATAGACAAAGT) was added to the *LuxS* gene between 303 bp and 304 bp and designated as Psa24 (Figure 5b). As shown in Figure 5c,d, the fluorescence intensity levels at these three intervals were 362, 810, and 1821 for Psr23, and 229, 653, and 969 for Psa24. As the control group, the fluorescence intensities of the original P*shuttle-09* were 1355, 1361, and 1594, respectively. Without xylose, the expression efficiencies of these two modified promoters (Psr23 and Psa24) were found to be limited by XylR based on the observation of a decrease in fluorescence intensities (26.71% and 16.9%, respectively). In comparison, the fluorescence intensity of cells containing Psr23 was greater than the control group, and a high expression efficiency was identified after an induction for 8h, whereas the expression efficiency of cells with Psa24 was still low even with the inducer.

## 3. Discussion

**Figure 6:**
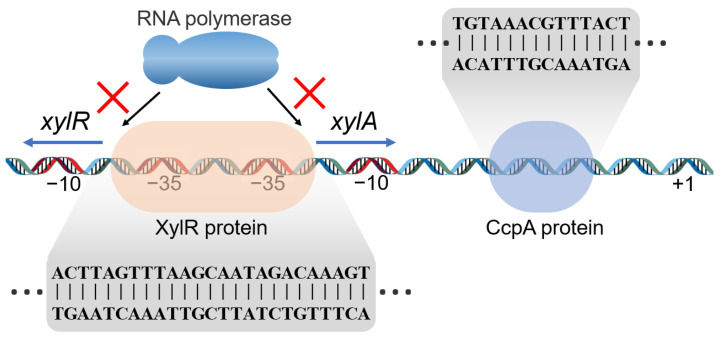
Construction of a xylose operon from *P. polymyxa*.

To improve the possible application potential of *P. polymyxa* by identifying promising expression elements, a xylose-inducible expression system was constructed and verified in this study. On the basis of the previously reported xylose operon in other *Bacillus* spp., the xylose operon candidate in *P. polymyxa* was compared against those of other bacterial taxa. The results reveal that those strains shared a similar basic bidirectional start promoter and regulatory mechanism. The construction of a xylose operon is shown in Figure 6. However, the *P. polymyxa* xylose operon indicated a difference in the length (135 bp) of the bidirectional promoters, which were 241 bp and 224 bp long in *B. subtilis* 168 and *B. licheniformis* ATCC14580 xylose operons, respectively [31]. This observation further suggested a variation in the structure of the xylose operons between different strains. In general, the XylR binding site was localized between the −10 region and the ribosome binding site (RBS) in *B. subtilis*, *B. licheniformis,* and *B. coagulans.* In *P. polymyxa,* however, the XylR binding site spanned both −35 regions from P*xylAB* to P*xylR*. In comparison, the regulator binds the −35 box more efficiently than the UTR region [38]. In addition, the transcription molecular mechanisms were thereafter different in accordance with the difference in the structure. In *Bacillus* spp., the XylR protein controls P*xylAB* transcription via a roadblock mechanism, whilst it sterically hinders RNA polymerase access to the P*xylAB* promoter in *P. polymyxa* [39]. The XylR also controls P*xylR* by preventing RNA polymerase from binding to the promoter. In this manner, the entire operon system is muted to avoid extra energy costs. Another issue that requires attention is the positions of the cre site in the promoter. *Bacillus* spp. has a typical cre sequence of TGWNANCGNTNWCA [40], while degenerate binding sites have the sequence TGNAANCGNNNNCN [41]. This discrepancy was used as a search query for putative cre elements in the *P. polymyxa* xylose operon. In the *B. subtilis* XylA operon, a cre was located at +36 bp [42], and the deletion of this cre site achieved a decrease in glucose repression from 13-fold to 2.5-fold. In *B. licheniformis*, a cre was localized at the core of the xylose operon. In plasmid-based systems, CCR also reduces expression. A mutation in the cre site may inhibit transcription regulation by CcpA [43]. For *P. polymyxa*, the putative cre site TGTAAACGTTTACT at the position between the −10 region and the RBS was detected herein, which differed from those of the other bacterial xylose operon systems. The difference gives the promoter the potential to be regulated by glucose and the possibility of relieving inhibition by modification.

A xylose-inducible promoter that functions in *P. polymyxa* was then developed in this study after the above structural analysis. The corresponding functionality of the P*xyl* promoter was further verified through the eGFP assay. In terms of the effect of temperature, the *P. polymyxa* growth rate was higher at 30 °C than at 37 °C, whereas the cellular fluorescence intensity was lower at 30 °C than at 37 °C. The optimum growth temperature for *P. polymyxa* was 30 °C, while the optimum PP*xyl* expression temperature was 37 °C. For optimum *P. polymyxa* fermentation, incubation should be performed at 30 °C. Subsequently, the induction was conducted at 37 °C after the appropriate biomass was attained. All xylose operons from other bacterial species used in this study were verified to work in *P. polymyxa*, although they showed different expression efficiencies. All xylose operons from other bacterial species used in the present study were verified to work in *P. polymyxa*, although they showed different expression efficiencies. Brito [13] reported a similar result and found that the xylose operon from *B. methanolicus* functioned in both *P. larvae* and *P. polymyxa*. However, as expression elements may not be adapted to a foreign host, operons from other bacterial species may have different promoter activities. In this study, the expression efficiency of the xylose operon from *B. subtilis* was extremely low in *P. polymyxa*, despite the fact that it is widely used as an expression system owing to its high expression level in *B. subtilis*. Thus, xylose operon from *B. subtilis* seems not to be a suitable expression system in *P. polymyxa*.

To further confirm the XylR protein binding site of *P. polymyxa,* the XylR protein from *P. polymyxa* was expressed and aligned with other ROK family proteins for the prediction of the XylR critical domain sequence. After alignment, the corresponding sequence was suggested to be ACTTAGTTT-AAGCAAT-AGACAAAGT, and the degenerate sequence ACTTWGTTT-AWSSNAT-AVACAAAGT was applied for all *Paenibacillus* spp. Due to the important roles played by the bases ACTTNG-CNAAGT at the leading and trailing ends in the XylR protein binding process, any alteration in these sequences was proposed to impede the binding. Overall, changes to the fifth A/A bases of the binding site were found to have less impact on the binding than changes to the other bases. Without entirely matching, the consensus sequence TTNGTTT-AAACNAA was determined for *B. subtilis* and applied to *B. licheniformis* and *B. megaterium* [44]. The second base C before TTNG was found to be critical for binding, which comprises part of the recognition site of the *N*-terminal effector subdomain position and super wing in the transcriptional regulator [45]. Similar phenomena in other *Paenibacillus* spp. were also observed, and C and G showed high affinity for this binding site [36]. The present study also demonstrated that the eighteenth base was functional except when it was mutated to A, which may be due to the specificity recognition of XylR. This disruption of the palindrome structure AAACNAA identified by HTH binding domain [46] may help repressors distinguish operons. Similar situations have been observed in the mannitol, mannose, sorbitol, and arabinose promoters P*mtlA*, P*manA*, P*gutA*, and P*araB*, which have similar binding site sequences but are controlled by their corresponding transcriptional regulators [47].

Considering the high expression efficiency of Pshuttle-09 in both B. subtilis and E. coli [48], the XylR binding site was transplanted to constitutive promoters with an observation of similar high expression efficiency in P. polymyxa. After a modification of the binding site on the *luxS* sequence to stop RNA polymerase transcription via the roadblock effect of the XylR protein, the modified Pshuttle-09 promoters Psr23 and Psa24 were inducible and had low leakage and high efficiency in response to inducers. However, the expression efficiency of Psa24 was significantly weaker than that of Psr23. A similar phenomenon was also reported in a previous study [49], in which it was suggested that the transcription might be harmed by A/G due to its effect on the expression intensity caused by the modification of 5′-UTR.

In summary, the xylose operon of *P. polymyxa* is highly efficient and could serve to construct a xylose-inducible system. This study is the first to present a sugar-inducible promoter from *P. polymyxa* that compensates for the regulatory expression element deficiency in this bacterium. With the construction of a free-expression system using the foregoing xylose operon, the endogenous xylose-inducible promoter was empirically demonstrated to be more efficient than the exogenous promoter from *B. subtilis*. A modified xylose-inducible P*shuttle-09* promoter was finally generated by transplanting the XylR binding site into a constitutive promoter after the identification and localization of the key bases of the regulatory site within the promoter via the comprehensive elucidation of the corresponding structure through EMSA.

## 4. Materials and Methods

### 4.1. Bacterial Strain and Culture Medium

The strains and plasmids used in the present study are listed in Table S1. The Top10 strain of *E. coli* was used in plasmid construction, while *E. coli* BL21(DE3) served to express the proteins. The *E. coli* transformants harboring plasmids modified by pHY300PLK were selected on Luria–Bertani (LB) agar plates supplemented with ampicillin (100 μg/mL). The *E. coli* harboring XylR expression vector was selected on LB agar plates fortified with kanamycin (30 μg/mL). The *P. polymyxa* transformants were selected on tryptic soy agar plates supplemented with tetracycline (20 μg/mL). The media used for the culture and activation of *E. coli* and *P. polymyxa* were LB medium (10 g/L tryptone, 5 g/L yeast extract, and 10 g/L NaCl) and tryptic soy broth (TSB) medium (15 g/L tryptone, 5 g/L soybean peptone, and 5 g/L NaCl; pH 7.2), respectively shaken at 200 rpm, and the *E. coli* was cultured at 37 °C while the incubation temperature of *P. polymyxa* was 30 °C.

### 4.2. Promoter-Function-Characterizing Plasmid Construction

A plasmid with a pHY300PLK backbone and harboring *eGFP* as a reporter gene was constructed to verify the validity and efficiency of the selected promoter. After the characterization of the promoter function for the constructed plasmid, the predict promoter PP*xyl* from *P. polymyxa* was inserted into the plasmid to test the corresponding efficiency within the bacterium. The function of PP*xyl* was determined, and xylose operons from *B. subtilis* and *B. licheniformis* were amplified and inserted into the plasmid to compare relative expression efficiency. The primers used are listed in Table 1. The *eGFP* gene was amplified with the eGFP-f/r primer pair and inserted into pHY300PLK plasmid at *Hin*dIII/*Sma*I, thereby generating the pHY-e plasmid. The upstream transcriptional regulator in the hypothetical xylose operon from *P. polymyxa* containing the promoter PP*xyl* (CP049783.1, 2818127-2818259) was obtained from *P. polymyxa* ATCC842 using the PPPxyl-f/r primer pair. The operons BS*xyl* (AL009126.3, 1891667-1891906) and BL*xyl* (CP000002.3, 3876057-3876247) were amplified from the genomes of *B. subtilis* 168 and *B. licheniformis* ATCC14580 using the BSPxyl-f/r and BLPxyl-f/r primer pairs, respectively. The operons were cloned into pHY-e by homologous recombination and the pHY-PPxyl, pHY-BSxyl, and pHY-BLxyl plasmids were generated from these clones. To construct plasmid pHY300-PS09, the *HapaII* promoter was obtained from pMA5 using HapaII-f/r, P*shuttle-09* was obtained using P09-f/r, and *xylR* (CP049783.1, 2816955-2818124) was obtained using 09xylR-f/r. All fragments are linked to pHY-e at HindIII by homologous recombination. The Psr23 and Psa24 fragments were obtained using Psr23-f/r and Psa24-f/r from pHY300-PS09, and they were further introduced into pHY300-Psr23 and pHY300-Psa24, respectively. All plasmids were transferred into *P. polymyxa* to verify the corresponding expression efficiency.

### 4.3. P. polymyxa Competent Cell Preparation

*P. polymyxa* was activated in TSB medium at 30 °C with shaking at 200 rpm for 10 h. Then, 0.5 mL of medium containing activated *P. polymyxa* cells was transferred to 50 mL of solution A (TSB with 0.5 M sorbitol) and cultured (30 °C, 250 rpm) for 8 h until OD_656_ = 0.8–0.9. The bacterial suspension was centrifuged at 6000× *g* at 4 °C for 5 min, kept in an ice bath for 30 min, and resuspended four times in solution B (10% (*v*/*v*) glycerol with 0.5 M sorbitol and 0.5 M mannitol. The cells were then resuspended in 1200 μL of solution B, divided into 20 equal parts, and stored at −80 °C until the subsequent experiments.

### 4.4. P. polymyxa Transformation

Plasmids with xylose operons were introduced by electrotransformation into *P. polymyxa*. Then, 4–8 μg of plasmid was mixed with 60 μL of *P. polymyxa* competent cells. The suspension was kept in an ice bath for 5 min, and the competent cells were transferred to a precooled 0.1 cm Gene Pulser cuvette and kept on ice for another 5 min. The electroporation conditions were 2000 V and 5 ms. Then, 1 mL of recovery medium (LB medium with 0.5 M sorbitol and 0.38 M mannitol) was added to the Gene Pulser cuvette immediately after the shock was terminated. After 8 h of recovery at 30 °C under 200 rpm shaking, the cells were applied to plates containing the corresponding antibiotics.

### 4.5. eGFP Fluorescence Measurement

The recombinant *P. polymyxa* strains harboring the plasmids were cultured in TSB medium at 30 °C and incubated at 200 rpm for 12 h. Then, a 5% (*v*/*v*) of the culture solution was inoculated into fresh TSB medium. After 12 h of fermentation, xylose inducer was added to a concentration of 20 g/L, and the fluorescence intensity of the culture was measured every 2 h thereafter. The samples were centrifuged to obtain the cells, and the latter were washed twice with phosphate-buffered saline (pH 7.4). The samples were diluted to OD_656_ = 0.5. Then, 200 μL aliquots were placed in each well of a 96-well microtiter plate. The sample plate was checked with a TECAN-SparK Plate Reader (Tecan, Männedorf, Switzerland). The scanning program and fluorescence intensity calculation were derived from a previously published report [47].

### 4.6. Electrophoretic Mobility Shift Assay

The ROK family proteins predicted to be XylR transcriptional regulators were expressed by *E. coli*. BL21(DE3). The *xylR* (GenBank No. CP049783.1) was copied from the *P. polymyxa* genome using the xylR-f/r primer pair and cloned at the *Nhe*I-*Hin*dIII sites of pET28a. A 134-bp promoter was amplified by the PE-f/r primer pair and ligated into pMD19-T to construct a plasmid template and obtain DNA probes for the polymerase chain reaction (PCR)-based EMSA. The 5′-biotin-labeled primer and others were used to amplify the biotinylated fragments. All marked fragments were purified by 2% agarose gel electrophoresis. The remaining steps were implemented following the instructions of the Chemiluminescent EMSA Kit (Beyotime, Shanghai, China).

### 4.7. Reverse Transcription Quantitative PCR (RT-qPCR)

*P. polymyxa* was activated overnight in TSB medium to determine the effects of xylose on the predicted operon. The latter had the overlapping promoters P*xylR* and P*xylAB* to regulate the transcription and the expression of XylA and XylB. The cells were incubated for another 12 h after 5% cultures were transferred to TSB medium, following which 2% xylose, glucose, or water was added. Each culture was sampled every 4 h. The mRNA was extracted with a Simply P Total RNA Extraction Kit (BSC52S1; BIOER, Hangzhou, China), and the cDNA was generated and amplified with a Prime Script RT Kit (TaKaRa Bio Inc., Kusatsu, Shiga, Japan). The genes *xylR*, *xylA,* and *xylB* were amplified with xR-F/xR-R, xA-F/xA-R, and xB-F/xB-r, respectively. The 16S housekeeping/internal reference gene was amplified with 16sF/R. The reaction conditions of the RT-qPCR were determined using a Universal YSBR qPCR Master Mix (Vazyme Biotech, Nanjing, China). The RT-qPCR was performed in a qTOWER2.0 thermal cycler (Analytik Jena, Jena, Germany).

## Figures and Tables

**Table 1:** Primers used to obtain fragments or construct plasmids.

Primers	Sequences (5’-3’)
16S-f	CCGCACAAGCAGTGGAGT
16S-r	TACCCAACATCTCACGACACGA
xA-f	GGAACACGGCGAACATGTTTAG
xA-r	CAGCAGTGTTTCGTAGCCTTCAC
xB-f	GGAGAAGCTAAATCAGGAGCAG
xB-r	GTGATGCTAACGTGCCACTG
xR-f	CGATACCGAGCACAAAGC
xR-f	CCACCGTGTCCAACCTG
xylR-f	CTAGCTAGCATGAATGTCACTGGCGATCAGGCG
xylR-r	CCCAAGCTTTAAAGACACGCGTACTCGCCCCA
eGFP-f	CCCAAGCTTATGGGTCGCGGATCCATGGTAG
eGFP-r	TCCCCCGGGTCACACGTGGTGGTGGTGGTG
BSPxyl-f	ATGGAAAAACGCTTTGCCCATTACATTGTAATCATGTCCAGAAAATGATC
BSPxyl-r	ACCATGGATCCGCGACCCATGTGATTTCCCCCTTAAAAATAAAT
BLPxyl-f	ATGGAAAAACGCTTTGCCCATTAAAATCTCTCGTTCATAAACCGTTCCA
BLPxyl-r	ACCATGGATCCGCGACCCAT TCCGATCTCCCCCTTCAC
PPPxyl-f	ATGGAAAAACGCTTTGCCCATTATAAAGACACGCGTACTCGC
PPPxyl-r	ACCATGGATCCGCGACCCATTATAAGTTCCTCCTTTGTAGTAAACG
PE-f	TTATAAAGACACGCGTACTCGC
PE-r	TATAAGTTCCTCCTTTGTAGTAAACGTTTACA
PxylABf(5′-biotin)	GCGCGGATCTTCCAGAGAT
PWS-f	GGTTATTTTCACTTCCTGTTGATGTAAT
Clip 1-f	TGATATTATATCATAAAAACAAACTTAGTTTAAGCAATAGACAAAG
Clip 2-f	TTAAGCAATAGACAAAGTTTCTTGGC
Clip 3-f	CTAGAATCGTTTTTGTAAACGTTTACTACA
Clip 4-f	AAGGAGGAACTTATAATGAATCTCTGG
BST-f	ACTTAGTTTAAGCAATAGACAAAGTCTTTTGG
DTE-f	GTTTAAGCAATAGACAAAGTCTTTTGGC
1T-f	TCTTAGTTTAAGCAATAGACAAAGATTCTTGGCT
1C-f	CCTTAGTTTAAGCAATAGACAAAGGTTCTTGGCT
1G-f	GCTTAGTTTAAGCAATAGACAAAGCTTCTTGGCT
2T-f	ATTTAGTTTAAGCAATAGACAAAATTTCTTGGCT
2A-f	AATTAGTTTAAGCAATAGACAAATTTTCTTGGCT
2G-f	AGTTAGTTTAAGCAATAGACAAACTTTCTTGGCT
3C-f	ACCTAGTTTAAGCAATAGACAAGGTTTCTTGGCT
3G-f	ACGTAGTTTAAGCAATAGACAACGTTTCTTGGCT
3A-f	ACATAGTTTAAGCAATAGACAATGTTTCTTGGCT
4C-f	ACTCAGTTTAAGCAATAGACAGAGTTTCTTGGCT
4G-f	ACTGAGTTTAAGCAATAGACACAGTTTCTTGGCT
4A-f	ACTAAGTTTAAGCAATAGACATAGTTTCTTGGCT
5C-f	ACTTCGTTTAAGCAATAGACGAAGTTTCTTGGCT
5G-f	ACTTGGTTTAAGCAATAGACCAAGTTTCTTGGCT
5A-f	ACTTTGTTTAAGCAATAGACTAAGTTTCTTGGCT
6T-f	ACTTATTTTAAGCAATAGAAAAAGTTTCTTGGCT
6A-f	ACTTAATTTAAGCAATAGATAAAGTTTCTTGGCT
6C-f	ACTTACTTTAAGCAATAGAGAAAGTTTCTTGGCT
17C-f	ACTTAGTTTAAGCAATACACAAAGTTTCTTGGCT
17A-f	ACTTAGTTTAAGCAATAAACAAAGTTTCTTGGCT
17T-f	ACTTAGTTTAAGCAATATACAAAGTTTCTTGGCT
-1T-f	TACTTAGTTTAAGCAATAGACAAAGTATCTTGGCT
-1C-f	CACTTAGTTTAAGCAATAGACAAAGTGTCTTGGCT
-1G-f	GACTTAGTTTAAGCAATAGACAAAGTCTCTTGGCT
-2T-f	TAACTTAGTTTAAGCAATAGACAAAGTTACTTGGC
-2C-f	CAACTTAGTTTAAGCAATAGACAAAGTTGCTTGGC
-2G-f	GAACTTAGTTTAAGCAATAGACAAAGTTCCTTGGC
HapaII-f	GATGGCGCATTGTGACGATCAAGCTTTTTTGAGTGATCTTCTCAAAAAATACTACC
HapaII-r	CATATGTAAATCGCTCCTTTTTAGGTGGC
P09-f	GATCGTCACAATGCGCCATCA
P09-r	ACCATGGATCCGCGACCCATGGATCCCACTTTATGGACGCCG
09xylR-f	GGAGCGATTTACATATGAATGTTACTGGCGATCAGGC
09xylR-r	TATGGAAAAACGCTTTGCCCTTACAAAGATACACGTACACGCCCGAGA
Psr23-f	CTTAGTTTAAGCAATAGACAAAGTAATGCAGTAAAAGCGCCTTACGTCAGACAC
Psr23-r	CTTTGTCTATTGCTTAAACTAAGTGAAGGCATGTTTCCTCTCTCCCCT
Psa24-f	CTTAGTTTAAGCAATAGACAAAGTGTGGGATCCATGGGTCGCGGA
Psa24-r	ACTTTGTCTATTGCTTAAACTAAGTTTATGGACGCCGCAGTGTCTGACGTAAG

## Data Availability

The data for the current study are available from the corresponding author on request.

## Supplementary Materials

The following supporting information can be downloaded at: https://www.mdpi.com/article/10.3390/ijms23095024/s1.
