# Supplementary material for: Analysis of Xylose Operon from Paenibacillus polymyxa ATCC842 and Development of Tools for Gene Expression
Source: Int J Mol Sci. 2022 Apr 30;23(9):5024. doi: 10.3390/ijms23095024 (PMC9104551; doi:10.3390/ijms23095024)
Supplement: Supplementary file 1 [file ijms-23-05024-s001.zip › ijms-1684750-supplementary.pdf]

Analysis of xylose operon from *Paenibacillus polymyxa* ATCC842 and development of tools for gene expression

Authors

Zilong Wang [1,2], Yakun Fang [1,2], Yi Shi [1,2], Yu Xin [1,2], ZhengHua Gu [1,2], Ting Yang [3,4], Youran Li [1,2], Zhongyang Ding [1,2], Guiyang Shi [1,2], Liang Zhang [1,2,*], and Jianmin Liu [5]

Affiliations

[1] National Engineering Research Center for Cereal Fermentation and Food Biomanufacturing, Jiangnan University, Wuxi, Jiangsu, 214122, P.R. China

[2] Jiangsu Provincial Engineering Research Center for Bioactive Product Processing, Jiangnan University, 1800 Lihu Avenue, Wuxi, Jiangsu 214122, P R China

[3] Wuxi Food Safety Inspection and Test Center, Wuxi, Jiangsu, 214142, P.R. China

[4] Technology Innovation Center of Special Food for State Market Regulation, Wuxi, Jiangsu, 214142, P.R. China

[5] Shandong huishilai Biotechnology Co., Ltd, Jinan Shandong 250000, P.R. China

***Corresponding author: Liang Zhang**

*E-mail: zhangl@jiangnan.edu.cn

Tel: +86-13861707271

```
B.subtilis_xylR             ......VDIADQTFVKKVNQKLLLKEILKNSPISRAKLSEMTGLNKSTVSSQVNTLMKESMVFEIGQGQS    64
B._licheniformis_xylR       .................MNKALIFEQIIENGPVSRAKLSEITGLNKATVSSQVSSLLQKDIIYETGPGES    53
P._polymyxa_xylR            .....MNVTGDQALVKKLNKSIVLERIRLHAPLSRAQLSSQTGLNKATVSNLVAELITDGLVYETGLGES    65
l._monocytogenes_Lmo0178    SNAMDILRKGNKDLIKDINRYTVLNLIREKGEITRTEIAKKCDFGMSTLTYILDDLQQEGIILEGAETSS    70
Consensus                                     n       i       r            t       l       e     s

B.subtilis_xylR             SGGRRPVMLVFNKKAGYSVGIDVGVDYINGILTDLEGTIVLDQYRHLE.SNSPEITKDILIDMIHHFITQ   133
B._licheniformis_xylR       SGGRRPVMLKFNRKAGYAVGVDVGTNYIIVALTDLEGHLIEQFERTLD.EEDIQATEEALIELTGLAVDK   122
P._polymyxa_xylR            SGGRKPLMLLFNSRAGFVLGIEVSVQYIKGALTDLAGTIETELTLSLT.QHDPTFVMEQIRKLVQELMQA   134
l._monocytogenes_Lmo0178    TGGRRAKLVRFNKDYGFVVSVKVEEEQLLFALTDLNAEIIENTSIPFSSEKKPEEAIELIAKNVKKMCGN   140
Consensus                    ggr      fn   g      v        ltdl

B.subtilis_xylR             MPQSPYGLIGIGICVPGLID.KDQKIVFTPNSNWRDIDLKSSIQEKYN.VPVFIENEANAGAYGEKVFGA   201
B._licheniformis_xylR       IPPSPFGLTGIGVCVPGLVD.NERHVVFTPNKPIHLIPIKEKLEERFG.VPILIENEANAGAVGEKEYGE   190
P._polymyxa_xylR            TPPSPHGIVGIGLGVPGMVD.ETGTVLFAPNLGWEEVPLRQQLEAELG.LPVVVDNEANVGAQGELYYGM   202
l._monocytogenes_Lmo0178    RDMN..HLLGVGIAISGLVNRKKGTVIRSTMLGWENVALEAMLHAHFPDIPVYVDKNINCYTLAELWLGE   208
Consensus                            g g    g                                 p       n     e   g

B.subtilis_xylR             A....KNHDNIIYVSISTGIGIGVIINNHLYRGVSGFSGEMGHMTIDFNGPKCSCGNRGCWELYASEKAL   267
B._licheniformis_xylR       G....GQLEHAVFVSINTGIGLGILMNGKLFRGVQGFSGEAGHMSIHFDGPLCRCGNRGCWELYASEKAV   256
P._polymyxa_xylR            DGDHRQQVRDLVYISAGSGIGAGIIIDGKPYQGAWGYAGETGHMTIDWNGRLCSCGSRGCWELYASEKAY   272
l._monocytogenes_Lmo0178    G....KQSNNFATVSVGAGLGLSVVINRQIYYGAQGGAGEFGHTTIQPGGYKCHCGQKGCLEMYASEFYF   274
Consensus                                 s   g g           g  g  ge gh  i   g  c cg  gc e yase

B.subtilis_xylR             LKSL........QTKEKKLSYQDIINLAHLNDIGTLNALQNFGFYLGIGLTNILNTFNPQAVILRNSIIE   329
B._licheniformis_xylR       FSHY........AANSGAQLYETVKELADRGDPGMMETFERFGFHIGIGLLNILKTLNPDTIILRNTIVE   318
P._polymyxa_xylR            VAS.........TLKLPAQNTAELLPFAQQGEVNTLSVLDDIGRYLGVGITNIVNSLNPGMLIIGGPLAE   333
l._monocytogenes_Lmo0178    RNRGEELKEAYPTSELNDFHFDKVAKSARAGDEMATELMGKMGEYLGYGIRNIINTFNPEKVIIVGEGLH   344
Consensus                                              a              g   g g  ni    np   i

B.subtilis_xylR             SHPMVLNSMRSEVSSRVYSQLGNSYELLPSSLGQNAPALGMSSIVIDHFLDMITM.......         384
B._licheniformis_xylR       SYPSIVDAIKKTIASRSAAEALSNYHLKISTLGRTASALGMSSLVTERFLERFMNERF....         376
P._polymyxa_xylR            ARPWLEQSMRDVIDERALPYHRRQLQIRFSTLGSRSTMLGAAYAATAPFLGRVRVSL.....         390
l._monocytogenes_Lmo0178    HRDLFLTKIDEIASQNFFSGAGFETEITTTSLEDPAWLQGAALLVIHQLFQVPIYEEEQTLL         406
Consensus                                                  l       g
```

Figure S1. The ROK family protein aligned with LMO0178 and xylR from *B. subtilis* and *B. licheniformis*.

```
P._polymyxa_HY96-2        .TATAAGTTCCTCCTTTATAGTAAACGTTTACAAAAACGATTCTAGCACAG.CCAAGAAACTTTGTCTA    67
P._polymyxa_CF05          .TATAAGTTCCTCCTTTATAGTAAACGTTTACAAAAACGATTCTAGCACAG.CCAAGAAACTTTGTCTA    67
P._polymyxa_SQR-21        .TATAAGTTCCTCCTTTATAGTAAACGTTTACAAAAACGATTCTAGCACAG.CCAAGAAACTTTGTCTA    67
P._polymyxa_EB4_G3        .TATAAGTTCCTCCTTTATAGTAAACGTTTACAAAAACGATTCTAGCACAG.CCAAGAAACTTTGTCTA    67
P._polymyxa_ZF129         .TATAAGTTCCTCCTTTGTAGTAAACGTTTACAAAAACGATTCTAGCACAG.CCAAGAAACTTTGTCTA    67
P._polymyxa_2020          .TATAAGTTCCTCCTTTGTAGTAAACGTTTACAAAAACGATTCTAGCACAG.CCAAGAAACTTTGTCTA    67
P._polymyxa_DSM_36        .TATAAGTTCCTCCTTTGTAGTAAACGTTTACAAAAACGATTCTAGCACAG.CCAAGAAACTTTGTCTA    67
P._polymyxa_ZF197         ..ATAAGTTCCTCCTTTGTAGTAAACGTTTACAAAAACGATTCTAGCACAG.TCAATAAACTTTGTCTA    66
P._polymyxa_SC2           .TATAAGTTCCTCCTTTGTAGTAAACGTTTACAAAAACGATTCTAGCACAG.CCAAGAAACTTTGTCTA    67
P._polymyxa_M1            .TATAAGTTCCTCCTTTGTAGTAAACGTTTACAAAAACGATTCTAGCACAG.CCAAGAAACTTTGTCTA    67
P._polymyxa_Sb3-1         .TATAAGTTCCTCCTTTGTAGTAAACGTTTACAAAAACGATTCTAGCACAG.CTGAGAAACTTTGTCTA    67
P._polymyxa_CJX518        .TATAAGTTCCTCCTTTGTAGTAAACGTTTACAAAAACGATTCTAGCACAG.CTGAGAAACTTTGTCTA    67
P._polymyxa_ATCC_15970    .TATAAGTTCCTCCTTTATAGTAAACGTTTACAAAAACGATTCTAGCACAG.CTAAAAGACTTTGTCTA    67
P._polymyxa_CR1           .TATAAGTTCCTCCTTTATAGTAAACGTTTACAAAAACGATTCTAGCACAG.CTAAAAGACTTTGTCTA    67
P._polymyxa_YC0573        .TATAAGTTCCTCCTTTATAGTAAACGTTTACAAAAACGATTCTAGCACAG.CCAAAAGACTTTGTCTA    67
P._polymyxa_E681          .TATAAGTTCCTCCTTTGTAGTAAACGTTTACAAAAACGATTCTAGCACAG.CCGAAAGACTTTGTCTA    67
P._polymyxa_J             .TATAAGTTCCTCCTTTGTAGTAAACGTTTACAAAAATGATTCTAGCACAG.CCAAAAGACTTTGTCTA    67
P._polymyxa_YC0136        .TATAAGTTCCTCCTTTGTAGTAAACGTTTACAAAAATGATTCTAACACAG.CCAAAAGACTTTGTCTA    67
Consensus                   ataagttcctccttt tagtaaacgtttacaaaaa gattcta cacag    a a actttgtcta

P._polymyxa_HY96-2        TTGCTTAAACTAAGTTTATTTTTATGATATAAT.AACGGGAATTACATCAACAGGAAGTGAAAATAAC    134
P._polymyxa_CF05          TTGCTTAAACTAAGTTTATTTTTATGATATAAT.AACGGGAATTACATCAACAGGAAGTGAAAATAAC    134
P._polymyxa_SQR-21        TTGCTTAAACTAAGTTTATTTTTATGATATAAT.AACGGGAATTACATCAACAGGAAGTGAAAATAAC    134
P._polymyxa_EB4_G3        TTGCTTAAACTAAGTTTATTTTTATGATATAAT.AACGGGAATTACATCAACAGGAAGTGAAAATAAC    134
P._polymyxa_ZF129         TTGCTTAAACTAAGTTTATTTTTATGATATAAT.AACGGGAATTACATCAACAGGAAGTGAAAATAAC    134
P._polymyxa_2020          TTGCTTAAACTAAGTTTATTTTTATGATATAAT.AACGGGAATTACATCAACAGGAAGTGAAAATAAC    134
P._polymyxa_DSM_36        TTGCTTAAACTAAGTTTATTTTTATGATATAAT.AACGGGAATTACATCAACAGGAAGTGAAAATAAC    134
P._polymyxa_ZF197         TTGCTTAAACTAAGTTTATTTTTATGATATAAT.AACGGGAATTACATCAACAGGAAGTGAAA.TAAC    132
P._polymyxa_SC2           TTGCTTAAACTAAGTTTATTTTTATGATATAAT.AACGGGAATTACATCAACAGGAAGGGAAA.TAAC    133
P._polymyxa_M1            TTGCTTAAACTAAGTTTATTTTTATGATATAAT.AACGGGAATTACATCAACAGGAAGGGAAA.TAAC    133
P._polymyxa_Sb3-1         TTGCTTAAACTAAGTTTATTTTTATGATATAAT.AACGGGAATTACATCAACAGGAAGGGAAA.TAAC    133
P._polymyxa_CJX518        TTGCTTAAACTAAGTTTATTTTTATGATATAAT.AACGGGAATTACATCAACAGGAAGGGAAA.TAAC    133
P._polymyxa_ATCC_15970    TTGCTTAAACTAAGTTTGTTTTTATGATATAAT.ATCAGGAATTACACCAACAGGAAGTGAA......    128
P._polymyxa_CR1           TTGCTTAAACTAAGTTTGTTTTTATGATATAAT.ATCAGGAATTACACCAACAGGAAGTGAA......    128
P._polymyxa_YC0573        TTGCTTAAACTAAGTTTGTTTTTATGATATAAT.ATCAGGAATTACACCAACAGGAAGTGAA......    128
P._polymyxa_E681          TTGCTTAAACTAAGTTTGTTTTTATGATATAAT.ATCAGGAATTACACCAACAGGAAGTGAAT.TAAC    133
P._polymyxa_J             TTGCTTAAACTAAGTTTGTTTTTATGATATAAT.ATTAGGAATTACACCAACAGGAAGTGAAT.TAAC    133
P._polymyxa_YC0136        TTGCTTAAACTAAGTTTGTTTTTATGATATAAT.ATCAGGAATCACACCAACAGGAAGTGAAT.TAAC    133
Consensus                 ttgcttaaactaagttt tttttatgatataat a   ggaat aca caacaggaag gaa
```

Figure S2. Full sequence alignment of xylose promoter from *P. polymyxa.*

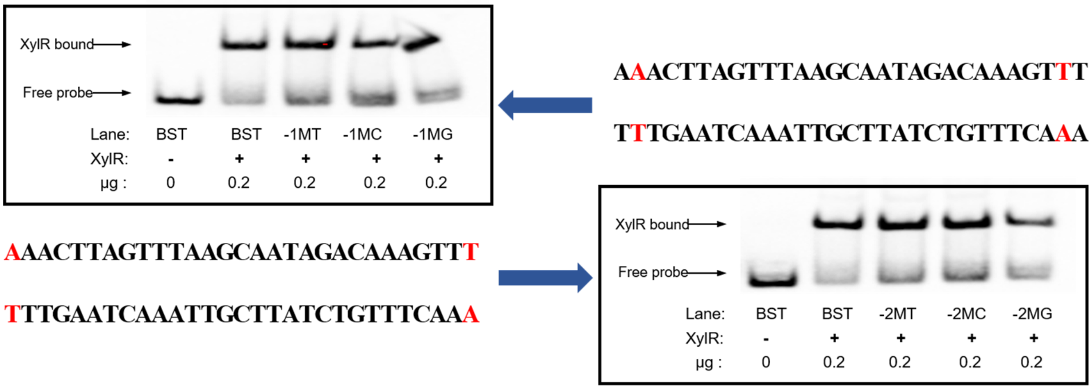

Figure S3. EMSA result of mutation bases out of the binding site.

Table S1. Bacterial strains and plasmids used in this study.

Strain or plasmid	Description	Reference
Strains		
E. coli Top10	F-, mcrAΔ(mrr-hsd RMS-mcrBC), φ80, lacZΔM15, △lacX74, recA1, araΔ139Δ(ara-leu)7697, galU, galK, rps, (StrR) endA1, nupG	lab collection
E. coli BL21(DE3)	F-, ompT, hsdS (rBB-mB-), gal, dcm(DE3)	lab collection
DE3-28aPPxylR	*E. coli* BL21(DE3), harboring pET28a-PPxylR	this study
B. subtilis 168	Wild-type	lab collection
B. licheniformis ATCC14580	Wild-type	lab collection
P. polymyxa ATCC842	Wild-type	CGMCC
PP-pHY	*P. polymyxa* ATCC842 harboring pHY-e	this study
PP-BSxyl	*P. polymyxa* ATCC842 harboring pHY-BSxyl	this study
PP-BLxyl	*P. polymyxa* ATCC842 harboring pHY-BLxyl	this study
PP-Pxyl	*P. polymyxa* ATCC842 harboring pHY-PPxyl	this study
PP-PS09	*P. polymyxa* ATCC842 harboring pHY300-PS09	this study
PP-Psr23	*P. polymyxa* ATCC842 harboring pHY300-Psr23	this study
PP-Psa24	*P. polymyxa* ATCC842 harboring pHY300-Psa24	this study
Plasmids		
pMD19-T-simple	*E. coli* cloning vector, ApR	TaKaRa
PPxyl-T	pMD19-T-simple derivative with xylose promoter from *P. polymyxa* ATCC842	this study
pET28a	*E. coli* expression vector, KanR	lab collection
pET28a-PPxylR	pET28a derivative with xylR from *P. polymyxa* ATCC842	this study
pHY300PLK	E. coli/Bacillus shuttle vector, ApR/TetR	lab collection
pHY-e	pHY300PLK derivative with eGFP	this study
pHY-BSxyl	pHY-e derivative with xylose promoter from *B. subtilis* 168	this study
pHY-BLxyl	pHY-e derivative with xylose promoter from *B. licheniformis* ATCC14580	this study
pHY-PPxyl	pHY-e derivative with xylose promoter from *P. polymyxa* ATCC842	this study
pHY300-PS09	pHY-e derivative with Pshuttle-09 promoter	this study

pHY300-Psr23	pHY-e derivative with Psr23 promoter	this study
pHY300-Psa24	pHY-e derivative with Psa24 promoter	this study
